# Tumor microenvironment: a prospective target of natural alkaloids for cancer treatment

**DOI:** 10.1186/s12935-021-02085-6

**Published:** 2021-07-20

**Authors:** Yanming Luo, Shuangshuang Yin, Jia Lu, Shiyue Zhou, Yingying Shao, Xiaomei Bao, Tao Wang, Yuling Qiu, Haiyang Yu

**Affiliations:** 1 Tianjin State Key Laboratory of Component-Based Chinese Medicine, Tianjin University of Traditional Chinese Medicine, Tianjin, 301617 China; 2 School of Pharmacy, Tianjin Medical University, Tianjin, 300070 China

**Keywords:** Tumor microenvironment, Tumor development, Alkaloids, Traditional Chinese Medicine

## Abstract

Malignant tumor has become one of the major diseases that seriously endangers human health. Numerous studies have demonstrated that tumor microenvironment (TME) is closely associated with patient prognosis. Tumor growth and progression are strongly dependent on its surrounding tumor microenvironment, because the optimal conditions originated from stromal elements are required for cancer cell proliferation, invasion, metastasis and drug resistance. The tumor microenvironment is an environment rich in immune/inflammatory cells and accompanied by a continuous, gradient of hypoxia and pH. Overcoming immunosuppressive environment and boosting anti-tumor immunity may be the key to the prevention and treatment of cancer. Most traditional Chinese medicine have been proved to have good anti-tumor activity, and they have the advantages of better therapeutic effect and few side effects in the treatment of malignant tumors. An increasing number of studies are giving evidence that alkaloids extracted from traditional Chinese medicine possess a significant anticancer efficiency via regulating a variety of tumor-related genes, pathways and other mechanisms. This paper reviews the anti-tumor effect of alkaloids targeting tumor microenvironment, and further reveals its anti-tumor mechanism through the effects of alkaloids on different components in tumor microenvironment.

## Background

**Fig. 1:**
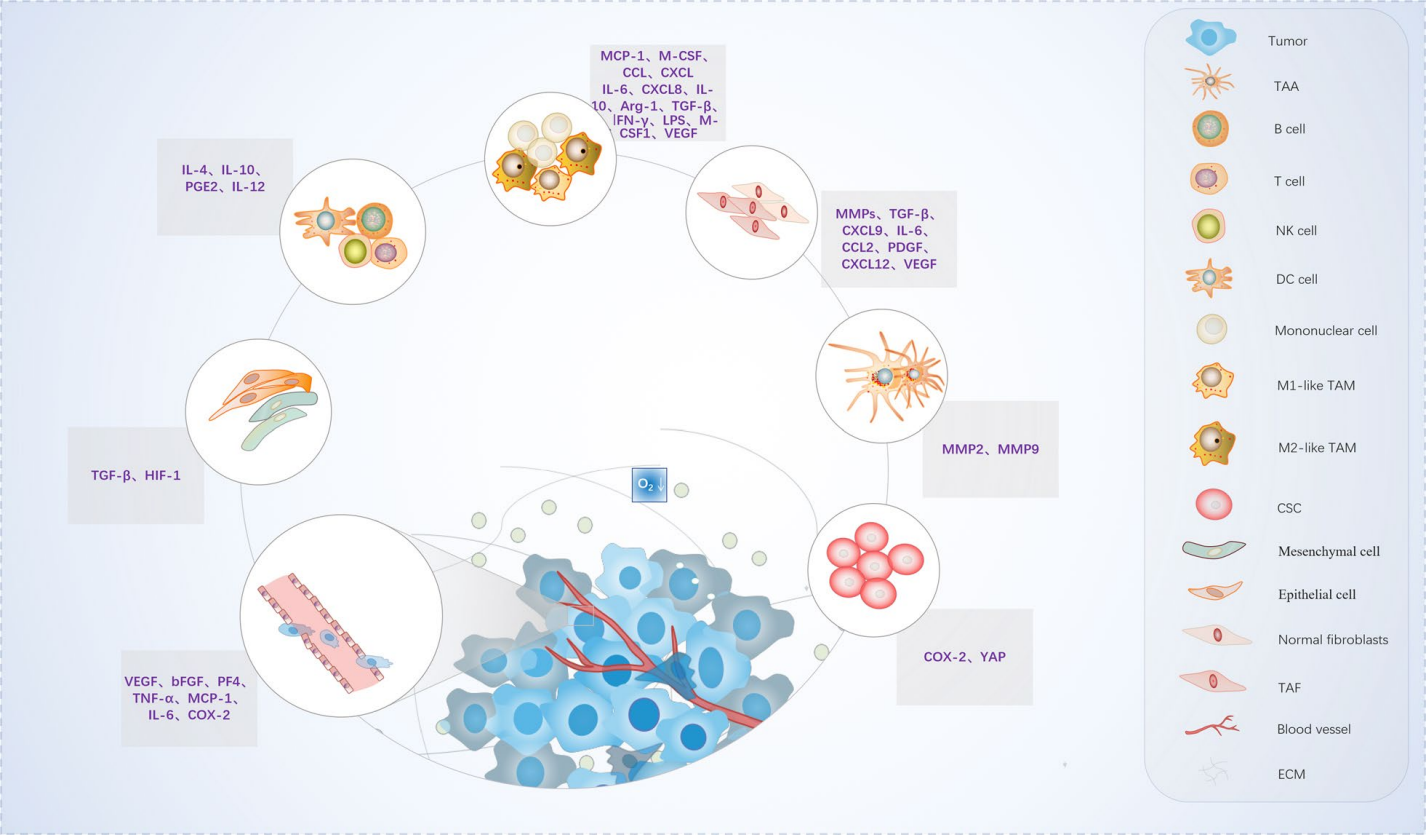
Brief introduction of tumor microenvironment (TME): tumor microenvironment consists of a variety of cell types, such as tumor cells, epithelial cells, immune cells, fibroblasts and stromal cells, accompanied by hypoxia and various secretory factors

Cancer as a major public health problem is one of the chief causes of death in the world [1]. Chemotherapy, radiotherapy and targeted therapy, as the existing methods for cancer treatment, are commonly used in clinical treatment. But their applications are greatly restricted due to various toxic side effects, such as drug resistance and cardiotoxicity. Although great efforts have been made to improve the understanding of biology and the development of cancer, there is still a lack of effective treatments, resulting in poor prognosis and high mortality [2–7]. Numerous studies have shown that the occurrence and development of tumor is a complex and multi-stage process, and closely related to TME (Fig. 1), On account of TME can reduce the permeability of drugs, endow tumor cells the ability to proliferate and anti-apoptosis, resulting in drug resistance and common modifications in disease morphology, which also has a significant influence on tumor prognosis and treatment efficiency [8, 9]. Therefore, shifting cancer treatments from inhibiting the growth of malignant tumor cells to TME and its complex interactions may be a new therapeutic strategy [10]. Natural products have shown possessing diverse bioactivities, and some of them or their derivatives display the potential of anti-cancer alone or in combination with conventional anti-cancer drugs or therapeutics. They may have the prospect of serving as excellent drugs leading to cancer prevention and anticancer drug discovery [11]. Alkaloids, as one of the natural products, are a kind of nitrogen-containing organic compounds with multiple complicated structure, widely exists in plenty parts of seeds, roots, stems, flowers, leaves, and fruits. Currently, numerous existing clinical medicines, especially anti-cancer drugs, derived from alkaloids and their derivatives. For instance, Paclitaxel and Camptothecin analogues are widely used in the treatment of various cancers [12–16]. A high concentration of paclitaxel (PTX) is used as an anti-tumor chemotherapy but is toxic to immune cells. At lower concentrations, PTX was found able to stimulate the anti-tumor potentials of immune cells. Tang, W. et al. foud that paclitaxel derivative-loaded nanoparticles showed lower cytotoxicity toward bone marrow-derived macrophages (BMDMs) than free paclitaxel, up-regulating the CD11b expression in BMDMs. This nanoparticle polarized macrophages toward M1 and inhibited their M2 differentiation, both on phenotypic and functional levels we have also added them in the text. For instance, F10 is a new camptothecin derivative, via attached saturated carbon atoms to the 10-position of camptothecin to synthesized 10 new camptothecin derivatives from 10-HCPT and SN-38. Activities of the new compounds were evaluated both in vitro and in vivo, these findings indicate that F10 is a new antitumor agent that is orally-bioavailable, potent and low in toxicity, and can thus serve as a new antitumor agent candidate for further studies [17, 18]. In addition, a lot of alkaloids have been extensively studied and proverbially reported because of their potent activities for the treatment of cancer. Previous studies have focused on systematically describing the distinct properties and diverse cellular functions of alkaloids, uncovered their important part in regulating proliferation, autophagy, metastasis, invasion, and radioresistance of tumor cells [19–21]. However, recently studies have drawn great interest from researchers to investigate the impact of TEM of alkaloids.

In this review, we comprehensively summarize the recent progress on the TME of alkaloids in various cancers, also document the molecular mechanisms of their anticancer activity, and focus on the specific cells of the TME during cancer progression (The mechanism regulated by alkaloids are showing in Table 1). Additionally, we highlight therapeutic strategies targeting these specific TME, because it is encouraging to move from standard current therapeutics to targeting key counterparts of the TME as future methods of cancer therapy. We aim to provide useful insights as well as a basis for clinical studies to develop alkaloids or its derivatives as an anticancer drug by targeting TME.

**Table 1:** Alkaloids in the tumor microenvironment have distinct functions during tumorigenesis

TME	Related factor	Alkaloids and their action mechanism	References
CAFs	TGF-β、OPN、SDF-1、VEGFA、IL-6、CXCL12、CXCL2、CXCL5、CCL2	Conophylline decreased IL-6, IL-8, CCL2, and CXCL12 secreted by CAF to suppress CAF activity and proliferation. Agelastatin alkaloids inhibits transcription of OPN in mammary fibroblasts in TME which are associated with increased invasiveness in cancers	[30–33]
TILs	CD28,CD39,IFN-γ, PD-1、CD8^+^、CD4^+^、IL-2、IL-4、IL-5、IL-13、TGF-β、FOXP3	Homoharringtonine reduced IL-12 cytokine expression and enhanced B-cell 、T cell and NK cell activation. Caffeine reduces the immuno-suppressive within the tumor microenvironment of CLL by inhibiting PI3Kδ. Piperine suppress the TH1、Th2-mediated immune responses, including the STAT6/GATA3/IL-4 signaling pathway. Oxyatrine regulates DC-Treg system in TME by promoting the maturation of DC and mediate the differentiation of T cells into Treg cells	[47–54]
TAMs	M1 maker: iNOs、CD86、TNF-α、IL-12、IFN-γ M2 maker: Arg-1、TGF-β1、CD206、IL-4、IL-10	Piperine exerts its anti-inflammatory effects via inhibition of COX-2 and NF-κB in murine macrophages. Curine treatment reduced cytokine levels and the expression of iNOS in vitro cultures of macrophages stimulated with LPS. Berberine inhibits the activation of NLRP3 inflammatory bodies to promote the polarization of M1. Ephedrine Hydrochloride increased IL-10 and decreased proinflammatory cytokine expression in primary peritoneal macrophages and Raw264.7 cells. Sophoridine can up-regulate the expression of iNOS, IFN-γ, IL-12α and down-regulate the expression of Arg-1, CD206 and IL-10, and have an effect on the polarization of TAMs in gastric TME. Solanine A suppressed LPS or INF-γ activated macrophages by inhibiting NF-κB, ERK1/2, AKT and STAT1 signaling pathways. Homoharringtonine inhibits STAT3 to suppress growth of cancer cells and sensitize cancer cells to the antitumor drugs. Lappaconitine inhibits the production of NO, PGE2 and TNF- α by inhibiting NF-kB and MAPK signaling pathways. Paclitaxel skews TAMs towards an immunocompetent profile via TLR4, which might contribute to the antitumor effect of PCX	[53, 70–73, 76–82, 84, 85, 88, 90]
MSCs/CSCs	VEGF, TRAIL	Ptx-PLGA NP-primed MSCs had enhanced sustained Ptx release in the form of free Ptx and Ptx NPs. Ptx transfer from MSCs to glioma cells could induce tumor cell death. Berberine reduce SDF-1 protein level secreted by BMSCs in the microenvironment to inhibit AML cells migration. LCL-HHT-H-PEG have an inhibitory effect on MM RPMI8226 CD138-CD34-CSCs. MASM inhibits hepatic cancer stem-like cells and markedly reduces the number of surviving cancer stem-like cells in the tumors. Isoharringtonine had inhibitory effects on BCSCs in breast cancer cell lines via inhibition of the STAT3/Nanong pathway.Berberis libanotica Ehrenb extract were sufficient to remove the self-renewal ability of highly resistant CSCs	[97, 99–101, 108, 109, 111, 112]
TAAs	IL-4, GFAP	Stachydrine suppresses proliferation and colony formation in Pilocytic astrocytoma cells through downregulated CXCR4 transcription and enhancing IκBα-based NF-κB inhibition. Palmatine suppresses glutamine-mediated changes in GLI signaling in PCCs while induces apoptosis by inhibition of survivin to disrupt reciprocal interaction between PSCs and PCCs in the TME Conophylline reduced liver and pancreatic fibrosis by suppression of stellate cells	[30, 115, 119, 121]
Angiogenesis	VEGF	α-solanine treatment significantly reduce the expression of VEGF and endothelial cell tube formation Berberine prevents the expression of HIF-1 to inhibit tumor-induced angiogenesis in hypoxic gastric cancer cells、HCC cells and human umbilical vein endothelial cells. Evodiaminevia down-regulation of VEGF expression and inhibition of tumor microangiogenesis in CRC mice modle. Narciclasine inhibits angiogenic processes through activation of Rho kinase and downregulation of the VEGF receptor 2	[124, 126, 128–132]
EMT	E-cadherin、N-cadherin、Vimentin、Twist	Piperine reversed the biomarker expression of EMT, and inhibits colorectal cancer migratory and invasive capacities through STAT3/ Snail mediated EMT. Sanguinarine inhibits the expression of EMT markers and also impairs HIF-1α and TGF-β form a feed-forward loop induce EMT in HCC cells. Sinomenine Hydrochloride suppressed the activation of NF-κB and the expression of MMP-2/-9, triggered ER stress, reversed the exogenous EMT. Berberine induced EMT changes in colonic epithelial cells with decreased E-cadherin and increased vimentin and α-SMA expression Halofuginone inhibits phosphorylation of SMAD proteins in response to TGF-β. Neferine suppressed EMT through an upregulation of E-cadherin and downregulation of Vimentin, Snail and N-cadherin and TGF-β	[2, 8, 39, 134–142]
ECM	MMPs	Morphine reducts the circulating MMP-9 and u-PA through modulation of paracrine communication between cancer cells and non-malignant cells in the tumor microenvironment. Emetine regulates two major MAPKs, p38 and ERK.This leads to the selective down-regulation of MMP-2 and MMP-9	[3, 146, 147]
ROS	–	Koumine possesses the cytoprotective effects by suppressing production of ROS、caspase-3 activity and influencing the expression of Bax and Bcl-2. Capsaicin through ROS-JNK-CCAAT/enhancer binding protein homologous protein pathway to inhibit cell proliferation, metastasis and induce apoptosis. Sophoridine provoked the generation of ROS in pancreatic cancer cells to develop its anti-tumor effects. 5-pyrrolidine-coupled naphthyl dihydroisoquinoline alkaloid priority cytotoxicity to PANC-1 cells which proliferate rapidly in tumor microenvironment under hypoxia conditions to inhibiting the growth of tumor cell	[159–162]

## Role of alkaloids in TME

### Targeting cancer-associated fibroblasts (CAFs)

Under physiological conditions, fibroblasts are the main components of ligated matrix tissue and wrapped in the interstitial extracellular matrix. Fibroblasts are the main cell type in stromal cells and contribute to the main function of connective tissue, and also promote to regulate tissue immune response by recruiting immune cells and making them more sensitive to bacterial lipopolysaccharide [22]. Startlingly, CAFs ought to be regarded as an entirely different cell type compared with normal fibroblasts. It is a key factor of the TME which compose of a group of heterogeneous activated fibroblasts. CAFs are the primary member of tumor stromal and one of the largest non-malignant host cell groups found in TME of breast, pancreatic and prostate tumors. A great deal of scientific evidences shows that these CAFs are not only closely related to tumors, but also actively recruited into the tumorigenesis, where they can affect other types of cells in the TME, as well as regulate the survival and metastasis of tumor cells [23–25]. CAFs have been reported to be involved in cancer progression through the secretion of growth or pro-inflammatory factors, such as transforming growth factor-β (TGF-β), hepatocyte growth factor (HGF), and C–X–C motif chemokine ligand 12(CXCL12) [26]. CAFs regulate immunosuppressive tumor infiltrating lymphocyte (TIL) by affecting the secretion of interleukin-6 (IL-6) in the TME [27, 28]. CAFs excrete connective tissue growth factor and TGF-β, which is the main cytokine and closely associated with cancer metastasis and other malignant behaviors. Blocking the expression of connective tissue growth factor and TGF-β is a practical way to restrain tumor cell migration [2, 29].

Conophylline (CNP) is an alkaloid extracted from tropical plant leaves. According to research findings, CNP inhibits CAFs activity and proliferation, sequentially, suppressing the stimulating effects of CAFs on pancreatic cancer cells. Moreover, CNP strongly decreases various cytokines secreted by CAFs are involved in cancer progression, such as IL-6, IL-8, C–C motif chemokine ligand 2 (CCL2), and CXCL12. Meanwhile, other studies also indicate that CNP combined with anti-cancer drugs is a promising strategy for the treatment of refractory pancreatic cancer [30]. In tumor microenvironment, the oncogenic activity of the tumor cell-intrinsic osteopontin (OPN), the expression of programmed death ligand 1 (PD-L1) and the expansion of tumor associated macrophages (TAMs) are the core drivers of immune escape [31]. The Agelastatin alkaloids are potent modulators for cancer invasion and migration at non-cytotoxic doses through inhibiting transcription of OPN in mammary fibroblasts. Specifically, OPN transcription and secretion are induced by negative regulation of the Rac GTPase exchange factor Tiam1. Beyond that, down-regulation of fibroblast Tiam1 and up-regulation of fibroblast OPN in the TME increased invasiveness in human breast cancers [32, 33].

### Targeting tumor infiltrating lymphocytes (TILs)

Lymphocyte is a kind of white blood cells produced by lymphoid organs, which mainly exists in the circulating lymph in the lymphatic vessels, and serves as an important cellular component of the body's immune response function. Meanwhile, lymphocyte is the main executor of almost all immune functions of the lymphoid system, and is a front-line “soldier” in the fight against external infection and monitoring of cellular variation in the body. TME also involves inflammatory cells, but they serve for tumor cells, promoting proliferation, survival and migration, and serving as an indispensable participant in tumorigenesis [34–36]. The interaction between infiltrating lymphocytes and tumor cells plays a vital part in the formation of immune microenvironment [37]. TILs, according to their migration, surface molecules and functions, can be divided into tumor infiltrating T cells, tumor infiltrating B cells and natural killer cells. On the basis of the surface phenotype and functional characteristics, CD4 + T cells can be classified into Th1, Th2, Th9, Th17, Tfh and Tumor-associated regulatory T cell (Treg) based on the surface phenotype, as well as the functional characteristics [38, 39]. Tregs has an auxiliary role in tumorigenesis, and cytotoxic T lymphocyte-associated antigen-4(CTLA-4) and programmed cell death protein 1(PD-1) are highly expressed. Therefore, blocking CTLA-4 or PD-1 could invalidate the Treg system. Moreover, Tregs secrete immunosuppressive cytokines, such as IL-10, IL-35 and TGF-β. TGF-β can change the phenotype of natural killer cells and T cells, thus enhancing their killing ability to tumor cells [40]. In addition, Treg also affects adjacent immune cells, such as promoting the secretion of IL-10 by dendritic cells (DC), which stimulates the expression of E3 ubiquitin ligase MARCH-I in activated macrophages and inhibits the self-activation of DCs, thereby down-regulating the antigen presentation of MHC-II and CD4 + T cells to play an immunosuppressive role [41].

Stachydrine hydrochloride (SH) is the main constituent of L. Sibiricus. SH could increase the expression of TGF-β significantly, but reduce the expression of EGF, SH also could significantly reduced the pathological changes of prostate hyperplasia. Moreover, SH promotes the protein expression of IL-12 and IL-6 and inhibits the mRNA level of FOXP3, and the expression of phosphorylated IκBα, nuclear factor-κB(NF-κB), p65, JAK2 and signal transducer and activator of transcription 3(STAT3) in vivo [39, 42, 43].

Activated B cells secrete a variety of cytokines, including interleukin, chemokine, lymphotoxin and interferon. However, B cells required additional signaling-could be provided by the tumor microenvironmen-beyond activation to become cytokine producers. Thus, Tumor-educated B cells (TEBs) might play key roles in tumor progression due to its ability to enhance the migration and invasion of tumors. It is reported that TEB could dramatically increase the renal cell carcinoma (RCC) cell migration and invasion. Mechanically, TEBs activated IL-1β/HIF-2α signals in RCC cells could mediate the downstream Notch1 signaling pathway to promote metastasis [44]. In TME, DCs control the strength and quality of antigen-specific adaptive immune responses. According to reports, mechanisms by which the canonical Wnt signaling cascade in DCs regulates immune suppression, and the same pathway in tumors is associated with the evasion of anti-tumor immunity. And Wnt/β-catenin pathway in DCs can be targeted for successful cancer immunotherapy [45]. Plasmacytoid dendritic cells (pDC) produce high levels of interferon-α (IFN-α), which is the main medium of antiviral immunity. Interestingly, pDC is a double-edged sword in the anti-tumor immune response. In the TME of head and neck squamous cell carcinoma, a naturally occurring pDC subgroup (OX40 + pDC), expresses high level of OX40. OX40 + pDC possesses unique immunostimulatory phenotypes and cell-killing functions, and the ability to produce tumor antigen-specific CD8 + T cell responses in conjunction with conventional dendritic cells (cDC) [46]. DC-Treg system plays a leading role in TME immunosuppression. It has a series of effector T cells, including immunogenic differentiation cluster CD4 + helper T cells, cytotoxic CD8 + T cells, and especially tolerant Tregs, called DC-Treg system.

**Fig. 2:**
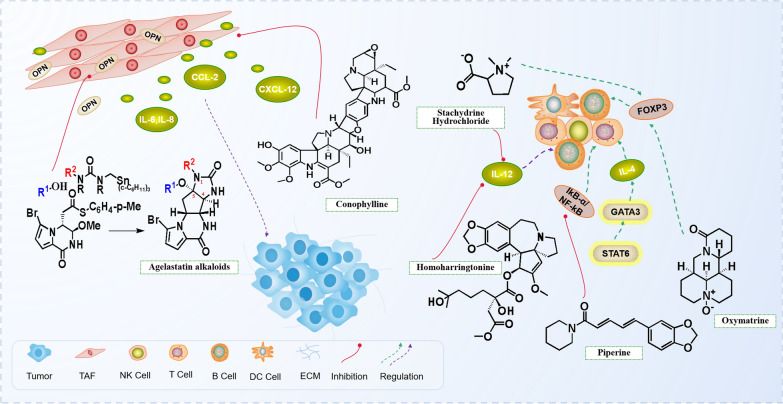
Alkaloids exert anti-tumor mechanism by targeting cancer-associated fibroblasts and tumor-related lymphocytes

Two alkaloids, Matrine (MT) and Oxymatrine (OMT) are extracted from the roots of *Sophora. Oxymatrine*. OMT regulates DC-Treg system in tumor microenvironment by promoting the maturation of DC and mediating the differentiation of T cells into Treg cells in vitro [47]. MT also decreases the expression of pro-inflammatory cytokines (TNF-α and IL-1β) in a dose-dependent manner. Homoharringtonine (HHT) is an alkaloid extracted from *Cephalotaxus*, and IL-12 is one of the key cytokines that promotes Th1 cells respond and induce T cells and NK cells to secrete IFN-γ. After HHT treatment, IL-12 is decreased in both subcutaneous and transgenic lung tumor models [48]. HHT also curbs IL-6/JAK1/STAT3 signal pathway in non-small cell lung cancer cell lines. The mechanism of its anti-myeloma may be the inhibition of AKT phosphorylation and several AKT target genes including NF-κB, XIAP, cIAP and Cyclin D1, the suppression of MCL1 protein synthesis, and the induction of apoptosis in chronic lymphocytic leukemia cells. HHT alone also significantly up-regulates the signal pathway of Notch, p53 and NF-κB [49–51]. Chronic Lymphocytic Leukemia (CLL) is related to T cell dysfunction. Activated CLL cells exist in the microenvironment of lymphoid tumors. Overcoming the immunosuppression induced by these cells may improve the anti-CLL immune response. Caffeine reduces the immunosuppressive activity of activated lymphocytes by inhibiting phosphatidylinositol kinase δ. These findings strengthen the fact that Caffeine may provide an effective therapy by reducing immunosuppression in the CLL tumor microenvironment [52]. Piperine impedes the progression of TNBS-induced colitis through regulating the nuclear factors of kappa light polypeptide gene enhancers in the B cell inhibitor-α/NF-κB signal pathway, to inhibit the overexpression of proinflammatory cytokines (TNF-α and ILs), cyclooxygenase-2(COX-2), colonic inducible nitric oxide synthase (iNOs), and oxidative-nitrous acid stress [53, 54] (Fig. 2).

### Targeting tumor associated macrophages (TAMs)

**Fig. 3:**
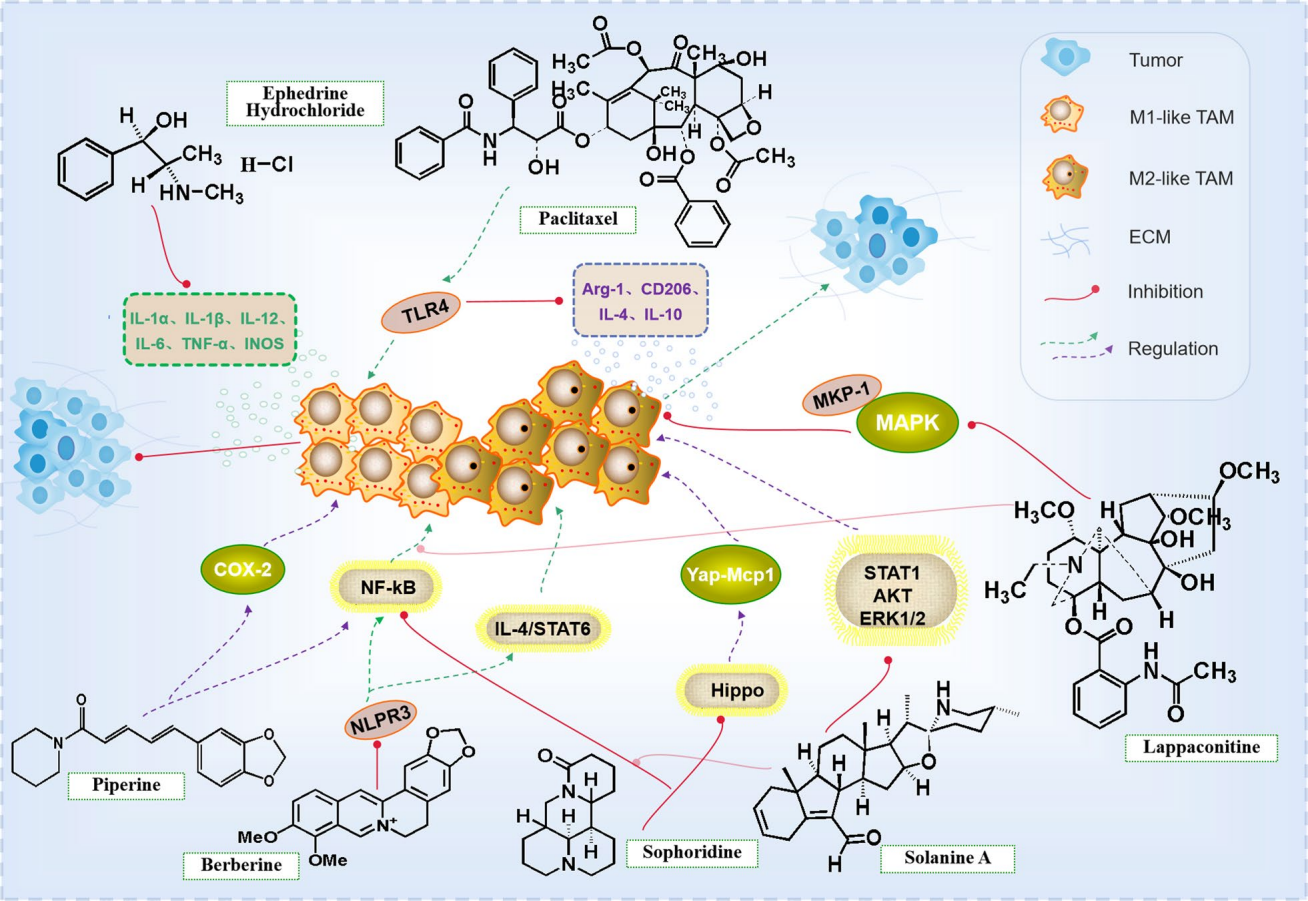
Alkaloids exert anti-tumor mechanism by targeting tumor-associated macrophages

In normal tissues, resident macrophages have specific phenotypes, such as microglia in the brain, Kupffer cells in the liver, alveolar macrophages in the lungs and peritoneal macrophages in the intestine. These macrophages may derive from embryonic precursors and maintain by self-renewal [55–60]. Different from macrophages, the plasticity and phenotypes of TAMs are according to different signals in TME, which also is the main cellular component and key regulator of tumor microenvironment. The infiltration of TAMs has been observed in various human cancers. M1 and M2, two main subtypes of TAMs, have opposite effects in TME. M1-type macrophages inhibit tumor survival, while M2-type macrophages support tumorigenesis. Repolarizing TAMs from M2 into M1 is a promising strategy for cancer treatment. The interaction between tumor and immune cells could regulate the malignant of tumor, immune cell infiltration and immune escape of TME in the tumor progression. The cytokines and chemokines secreted by this crosstalk occupy considerable position in the occurrence, development, and treatment of cancer [61–65]. TAMs display a continuum of different polarization states between tumoricidal M1 phenotype and tumor-supportive M2 phenotypes. When the ratio of M2/M1 is higher, it performs to accelerate tumor growth by promoting angiogenesis, immunosuppression and enhancing cancer cell invasion and metastasis, and vice versa [11, 66]. TAMs express PD-L1, which participates in the tumor microenvironment of immunosuppression. PD-L1 transmits structural negative signals to macrophages, resulting in cell phenotype of immunosuppressive elated [67]. Activated macrophages produce pro-inflammatory factors such as TNF-α, IL-1β, IL-6, iNOs and COX-2 [68]. Curine is a bisbenzylisoquinoline alkaloid, and it significantly inhibits the recruitment of neutrophils, TNF-α, IL-1β, IL-6, monocyte chemotactic protein (CCL2/MCP-1) and IL-4 in pleural lavage fluid, meanwhile lowers the expression level of cytokines and iNOs in macrophages stimulated by LPS [69]. Berberine (BBR) is an alkaloid extracted from Rhizoma Coptidis. BBR reduces the cell viability of MDA-MB-231 cell line of breast cancer with a dose-dependent manner. BBR not only decreases the secretion of pro-inflammatory cytokines, IL-1α, IL-1β, IL-6 and TNF-α, but also remarkably down-regulates the expression of NLR family pyrin domain containing 3(NLRP3), Caspase-1 and recruitment domain (ASC), which are the main components of NLRP3 inflammatory complex. BBR exerts its anti-tumor effect by inhibiting the activation of NLRP3 inflammatory bodies mediated by P2X7 [70]. The activation of NLRP3 inflammatory bodies is critical in the polarization of M1 macrophages [71]. NLRP3 activates multiple cell types by mediating NF-κB activation [72, 73]. When NLRP3 activation is inhibited, NF-κB and IL-4/STAT6 signal transduction will be obstructed, ultimately inhibiting M1 and M2 macrophage infiltration [74]. NF-κB is the main transcription factor regulating the immune function of tumor microenvironment, and it has the functions to convert the M2 to the M1 polarized TAM. Most importantly, it tilts the cytokine spectrum to the antineoplastic factor spectrum in the TME [75]. Piperine has the biological effects of anti-oxidation, anti- inflammation, anti-apoptosis and anti-ulcer. Piperine exerts its anti- inflammatory effects via inhibition of COX-2 and NF-κB in murine macrophages, and significantly reduces the mRNA expression level of iNOs, TNF-α, IL-1β, and IL-6 to regulate immunity [53, 76]. Ephedrine hydrochloride is a compound from ephedrine, derived from Ephedra sinica, which increases IL-10 and decreases pro-inflammatory cytokine (IL-6, TNF-a, IL-12 and IL-1β) expression in primary peritoneal macrophages and RAW264.7 cells [77]. Sophoridine is a quinolizidine monomer alkaloid from Sophora alopecuroides, who has multiple pharmacological effects, such as anti-tumor, anti-inflammatory, anti-arrhythmia, and anti-virus [78, 79]. Sophoride acts on macrophages and CD8^+^T cells to reshape the immune microenvironment of gastric cancer, and it also up-regulates the expression of iNOs, IFN-β, and IL-12α, and down-regulates the expression of Arg-1, CD206 and IL-10, exerting an effect on the polarization of TAMs in gastric tumor microenvironment and playing a stronger anti-inflammatory part. Sophoridine enhances the proliferation and cytotoxicity of CD8^+^T cells, and down-regulates the expression of CD8^+^T cellular markers PD-1, TIM-3 and LAG-3 by up-regulating the expression of granzyme-B, TNF-α and perforin of TAMs. What’s more, Sophoridine directly inhibits the cell viability of HepG2 by regulating PTEN/PI3K/AKT, Caspase-3/9 and Matrix metalloproteinase (MMP)-2/9 signal pathways. In addition, Sophoridine also inhibits the migration of macrophages by reducing the expression of CCR2 [80–82]. Sophoridine evidently activates Hippo and p53 signal pathways to inhibit the occurrence and development of lung cancer [78]. Hippo signal transduction in hepatocytes can maintain normal liver growth and inhibit macrophage infiltration during tumor microenvironment formation by inhibiting Yap-dependent MCP-1 expression [36]. IL-1β and TNF-α are important regulatory factors. Inhibition of TNF-α could prevent the formation of aneurysms, prevent matrix degradation, and barrier the expression of matrix metalloproteinases partly by blocking the activation of macrophages [83]. Solanine A is a steroidal alkaloid isolated from Solanum nigrum. Solanine A shows strong anti-inflammatory activity in LPS or IFN-γ activated macrophages and animal inflammatory models by inhibiting NF-κB, ERK1/2, AKT and STAT1 signaling pathways [84]. Lung cancer patients have a high level of IL-6, mutated epidermal growth factor receptor and TGF-β. Il-6 raises STAT3 activation by GP130/JAK pathway, and HHT, through GP130/JAK pathway, reversely inhibits IL-6-induced STAT3 tyrosine 705 phosphorylation and declines the protein expression of anti-apoptotic [49, 85]. In hepatocellular carcinoma patients, the silencing of SIRT4 in TAMs significantly modulates the alternating activation of macrophages to promote the development of hepatocellular carcinoma. The down-regulation of SIRT4 is related to the increase of macrophage infiltration and the high ratio of M2/M1 in the peritumor tissue of HCC. The increased MCP-1 is the major reason for the TAMs infiltration. We found that downregulation of SIRT4 in TAMs modulates the alternative activation of macrophages via the FAO-PPARδ-STAT3 axis and promotes macrophage infiltration by enhancing MCP-1 expression via the NF-κB pathway [86]. NF-κB and Mitogen-activated protein kinases (MAPKs), including the extracellular signal-regulated kinases (ERKs), c-Jun N-terminal kinases (JNKs), and p38-MAPKs, have been reported to be involved in macrophages polarization [87]. Lappaconitine is a natural compound with a C18-diterpene alkaloid skeleton and has a wide range of biological properties. It inhibits the production of NO, prostaglandin E2 (PGE2) and TNF-α by inhibiting NF-κB and MAPK signaling pathways, showing anti-inflammatory mechanism [88]. The activation of MAPKs and the degradation of NF-κB are the leading reasons for the transformation of M2-like to M1-like macrophages. STAT6 is also involved in the regulation of polarization of M2-type. Activated STAT6 directly bind to the promoters of some downstream genes, such as Fizz1 [89]. Paclitaxel (PCX) is a diterpenoid alkaloid with anti-cancer activity, which is widely used in the treatment of many kinds of solid tumors. PCX reprograms M2-like to M1-like phenotype in a TLR4-dependent manner, and regulates tumor-associated macrophages in breast cancer and melanoma mouse models [90] (Fig. 3).Randomized phase II study of second-line chemotherapy with the best available 5-fluorouracil regimen versus weekly administration of paclitaxel, severe gastrointestinal toxic effects were observed in only a few patients in the wPTX arm despite intestinal stenosis and/or ascites [91, 92]. Alstonia scholaris was reported to be a rich source of indole alkaloids, which exhibited remarkably bioactivities. scholaris has been registered as investigational new botanical drug (No. 2011L01436) and was approved for phase I/II clinical trials by China Food and Drug Administration (CFDA) [93].

### Targeting mesenchymal stem cells (MSCs)

Mesenchymal stem cells (MSCs) locate in most tissues, such as adipose tissue, skin, heart, kidney, or around blood vessels. MSCs have the signature characteristics of stem cells in terms of self-renewal and differentiation, which could differentiate into fibroblasts, adipocytes, osteoblasts, chondroblasts, blood vessels and perivascular structures. MSCs is not only an important part of bone marrow mesenchymal stem cells, but also an important part of tumor microenvironment [94]. MSCs contribute to the protection, regeneration and proliferation of hematopoietic stem cells and participate in the repair of tissue injury by creating regenerative microenvironment and differentiating into functional tissue-specific cells [95]. MSCs have the characteristics of low immunogenicity, rapid expansion in vitro, inherent pro-tumor and migration, and can be used as cell carriers for tumor therapy [96, 97]. Malignant glioma is a kind of primary brain tumor, which has the characteristics of high proliferation, invasiveness and poor prognosis. Although some advanced treatments are used, the prognosis is poor. It is reported that bone marrow mesenchymal stem cells can carry out tumor targeted gene therapy by carrying therapeutic genes, which may be a new promising treatment method [98].

The bone marrow mesenchymal stem cell (BMSCs) encapsulated by paclitaxel (PTX)-poly (dpene-lactide-glycolide) nanoparticles (PLGA) is an effective method for the treatment of gliomas. Therefore, the proposal of the incorporation of chemotherapeutic drug-loaded nanoparticles into bone marrow mesenchymal stem cells is feasible [97, 99]. Under hypoxia, MSCs can increase the expression of COX-2, promote the secretion of PGE2, and then activate YAP, to promote the proliferation of HCC cells and tumor growth. Blocking the communication between MSCs and cancer cells also can be used as a potential therapeutic target to inhibit the growth of cancer [100]. Berberine may partially inhibit the migration of primary acute myeloid leukemia (AML) cells by reducing the level of SDF-1 protein secreted by bone marrow mesenchymal stem cells in microenvironment and inhibiting HERG1 potassium channel of leukemic cells. It is speculated that Berberine can be used as an effective drug to prevent leukemia in the future [101].

### Targeting cancer stem cells (CSCs)

**Fig. 4:**
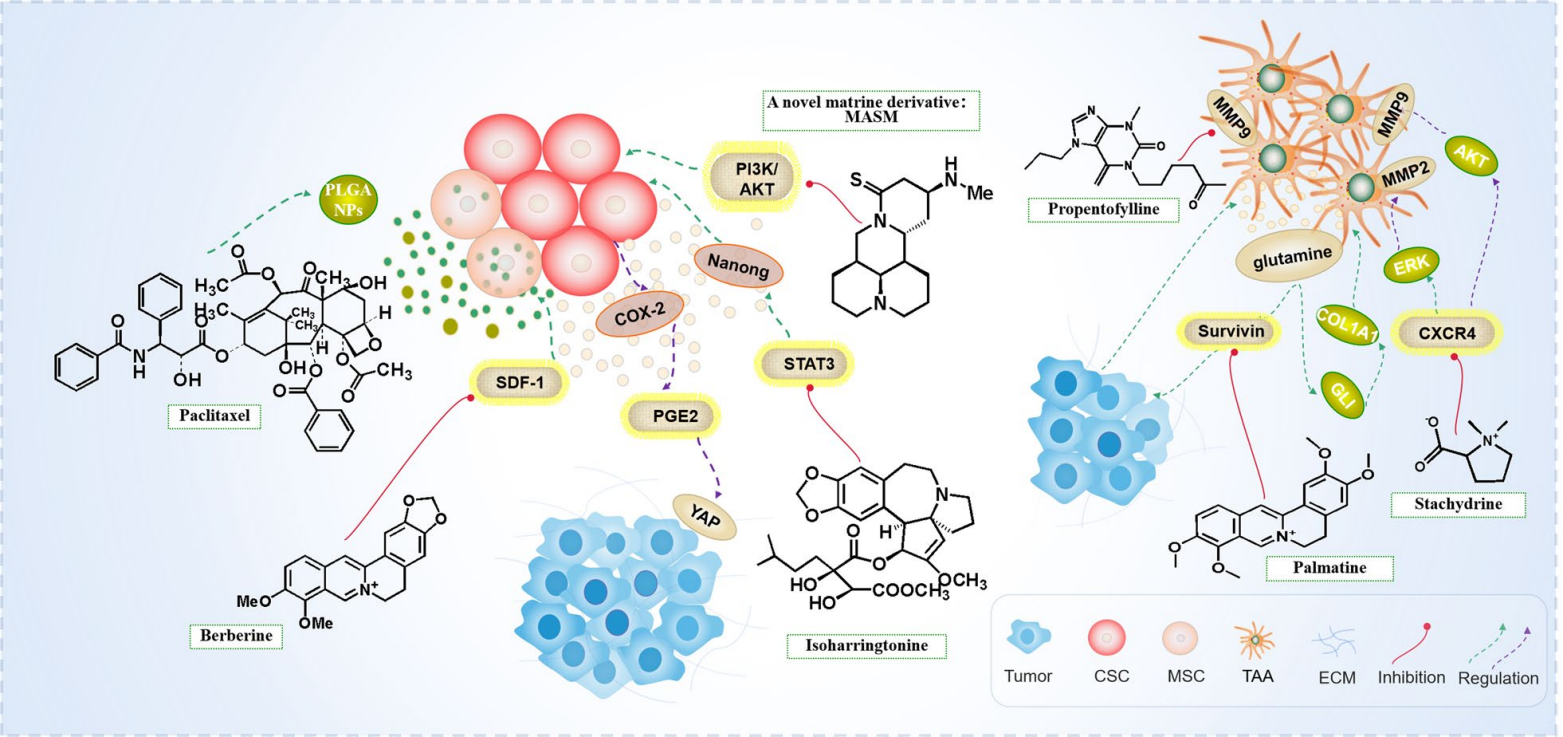
Alkaloids exert anti-tumor mechanism by targeting mesenchymal stem cells and tumor-associated astrocytes

In tumor tissue, cancer stem cells (CSCs) are a self-renewing cell group with high carcinogenicity. CSCs in tumor microenvironment plays an important role in tumorigenesis and development, to promote tumor growth, metastasis and drug resistance [102–106]. Accumulating evidence suggests intratumoral heterogeneity is a major ongoing challenge in the effective therapeutic targeting of cancer. However, there are a fraction of cells within a tumor termed CSCs being primarily responsible for this diversity resulting in therapeutic resistance and metastasis [107]. Multiple myeloma (MM) is a common hematological malignant tumor. PEGylated long-circulating liposomes containing HHT (LCL-HHT-H-PEG) have an inhibitory effect on MM RPMI8226 CD138^−^CD34^−^CSCs [108]. Matrine has shown chemopreventive potential against various cancers. A novel matrine derivative of matrine, (6aS,10S,11aR,11bR,11cS)-10-methylamino-dodeca hydro-3a,7a-diazabenzo(de) anthracene-8-thione (MASM) inhibits hepatic cancer stem-like cells by suppressing PI3K/AKT signaling pathways and markedly reduces the number of surviving cancer stem-like cells in the tumors [109]. Breast cancer stem cells (BCSCs) help with breast cancer progression, relapse, and treatment resistance. Isoharringtonine has inhibitory effects on BCSCs in breast cancer cell lines via inhibition of the STAT3/Nanong pathway [110]. *Berberis libanotica Ehrenb* (BLE) is a plant rich in alkaloids which may possess anti-cancer activity. And interestingly BLE extract has a major effect on CSCs. Three rounds of treatment with BLE extract are sufficient to remove the self-renewal ability of highly resistant CSCs [111, 112] (Fig. 4).

### Targeting tumor-associated astrocytes (TAAs)

Glioblastoma (GBM) is the most common and invasive brain cancer [113]. Astrocytes are the main components of GBM microenvironment, and astrocytes are the most common cells that interact with GBM cells among many cell types of microenvironment [114]. The astrocytes in Medulloblastoma Tumor TME are reversely differentiated from tumor granule neuron precursor (GNPs), the precursor of tumor granule neurons [115]. In the process of occurrence and development of GBM, tumor-associated astrocytes (TAAs) directly contact GBM cells, activate astrocytes to form reactive astrocytes, and promote tumor progression, proliferation and migration through a number of known signal pathways. Microglia secretes a variety of mediators that promote tumor growth and invasion, such as MMPs, MMP-2 and MMP-9, who are considered to be the key enzymes involved in glioma invasion [116, 117]. Astrocytes in tumor microenvironment can be targeted by the drug valprophylline. Propentofylline targets microglia rather than tumor cells. Astrocytes increase glutamate uptake through GLT-1 transporters, resulting in a decrease in glutamate available to CNS-1 cells and ultimately increasing apoptosis in CNS-1 cells. It also significantly inhibits the tumor growth of GBM rat model [117, 118]. Stachydrine(STA) is a compound extracted from *Leonuru heterophyllus*. Stachydrine dose-dependently suppresses proliferation and colony formation in Pilocytic astrocytoma cells with no such effect on normal astrocytes. STA also significantly increases the protein expression of TIMP-1 and TIMP-2, and decreases the protein expression of MMP-2 and MMP-9 [119, 120]. Reciprocal interaction between pancreatic stellate cells (PSCs) and cancer cells (PCCs) in the TME promotes tumor cell survival and progression to lethal. PCCs also promotes tumor progression by secreting a variety of cytokines and growth factors, including paracrine activation of pscs by Sonic Hedgehog. Palmatine is an isoquinoline alkaloid, which suppresses glutamine-mediated changes in GLI signaling in PCCs, resulting in the inhibition of growth and migration while inducing apoptosis by inhibition of surviving to disrupt reciprocal interaction between pancreatic stellate PSCs and PCCs in the TME [122] (Fig. 4).

### Targeting angiogenesis

**Fig. 5:**
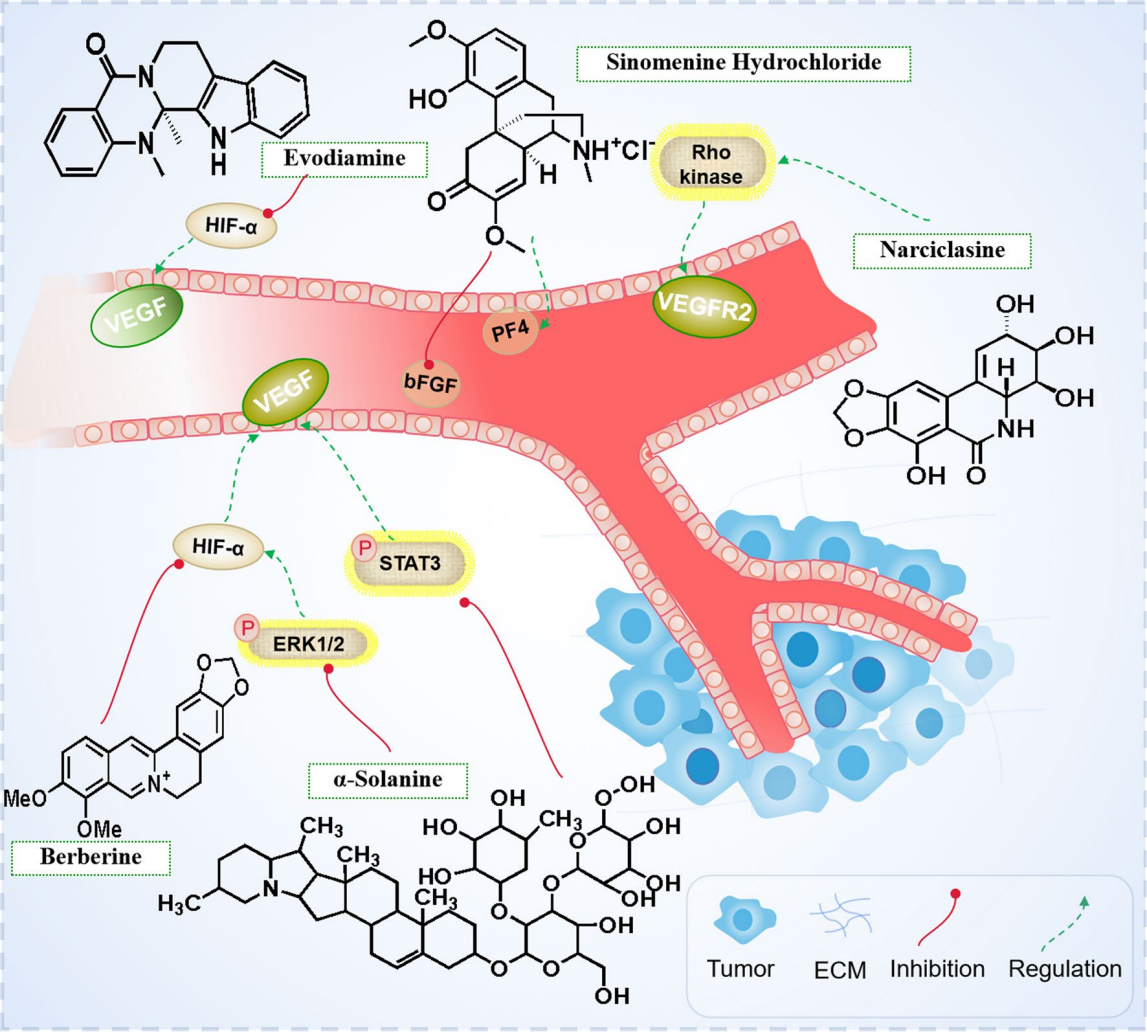
Alkaloids exert anti-tumor mechanism by targeting angiogenesis

Angiogenesis is extremely essential for the growth, metastasis and prognosis of malignant solid tumors. Vascular endothelial growth factor (VEGF) is one of the most effective angiogenic growth factors, and p-STAT3 is also a key nuclear transcription factor regulating all aspects of angiogenesis. α-Solanine, a trisaccharide glycoalkaloid, has anti-cancer effects on pancreatic cancer, liver cancer, breast cancer and melanoma cells [123, 124]. α-Solanine treatment significantly reduces the expression of VEGF and endothelial cell tube formation. α-Solanine inhibits VEGF expression, and affects ERK/AKT-Hypoxia-inducible factor-1α(HIF-1α)-VEGF and STAT3-VEGF signal pathways. α-Solanine also inhibits the mRNA expression of iNOs, COX-2, IL-6, TNF-α and IL-1β in liver [125, 126]. Berberine promotes apoptosis and anti-cancer effects on a variety of human cancer cells, and can inhibit the growth of many cancer cell lines, such as liver, lung, stomach, colon, skin, esophagus, brain, etc. Berberine prevents the expression of hypoxia inducible factor (HIF-1) in hypoxic gastric cancer (SC-M1) cells; HIF-1 is the key factor mediating tumor angiogenesis. HIF-1 inhibition is a key step for Berberine to inhibit tumor-induced angiogenesis. Berberine also inhibits tumor-induced angiogenesis in HCC cells and human umbilical vein endothelial cells [127]. Sinomenine is an alkaloid extracted from the traditional Chinese medicine *Sinomenine*. Sinomenine hydrochloride (SH) has a wide range of pharmacological effects, including anti-inflammation, immune regulation, anti-angiogenesis, anti-arrhythmia, and mild sedative and analgesic effects. The effect of SH on tumor vessels is due to its ability to restore the balance between pro-angiogenic factor (bFGF) and anti-angiogenic factor (PF4), which may have a great impact on the tumor microenvironment [128]. Evodiamine (Evo), an indolequinone alkaloid, is the major bioactive compound extracted from *Evodia rutaecarpa*. It may be via down-regulation of VEGF expression and inhibition of tumor microangiogenesis in colorectal cancer (CRC) mice model. EGFR-targeting Evo-encapsulated poly (amino acid) nanoparticles (GE11-Evo-NPs) dramatically down-regulate the expression of EGFR, VEGF, and MMP proteins, which may partially account for their inhibition of invasion and metastasis of CRC [129, 130]. Evodiamine also exerts its anti-hepatoma activity by inhibiting the activation of NF-κB and MAPK [131]. Narciclasine, a plant alkaloid from the Amaryllidaceae family, has extensively been characterized as anti-tumor compound. It mediates its anti-angiogenic effects in part by a RhoA-independent activation of the Rho kinase ROCK and reduces the de novo protein synthesis in endothelial cells by approx. As a consequence, Narciclasine diminishes the presence of proteins with a short half-life, such as the VEGF receptor 2, which is the basis for its anti-angiogenic effects [132] (Fig. 5).

### Targeting epithelial mesenchymal transformation (EMT)

**Fig. 6:**
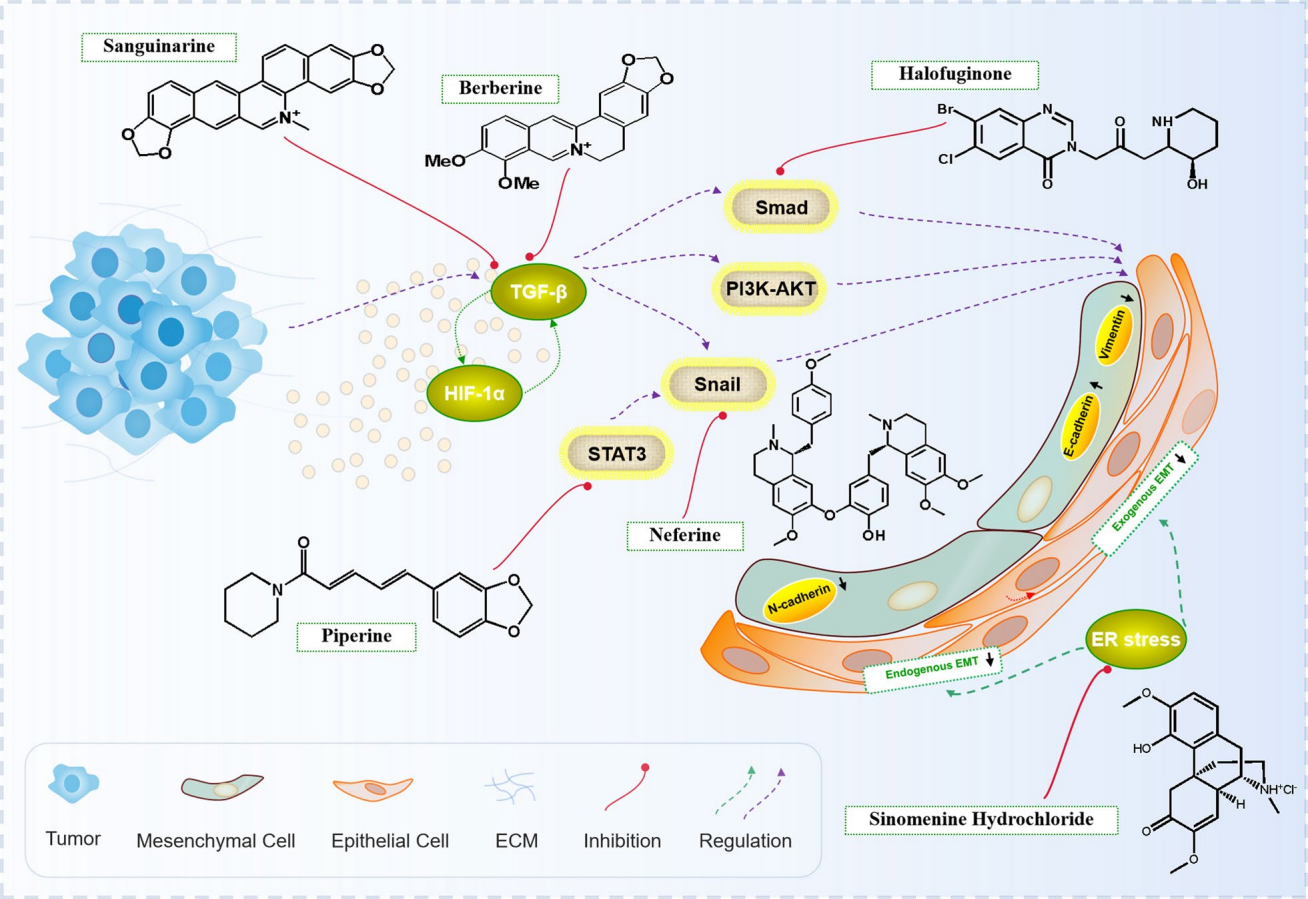
Alkaloids exert anti-tumor mechanism by targeting EMT

Stromal cells and cancer cells in TME produce a variety of inflammatory cytokines [133]. Especially in the process of tumor metastasis, although intimal metastasis is a complex process, hypoxia and excessive production of TGF-β in tumor microenvironment are undoubtedly important factors to induce intimal metastasis. During EMT, cancer cells acquire the interstitial characteristics of migration and invasion of the surrounding matrix because of the loss of cell-to-cell connection, cell–matrix contact and normal epithelial polarity [134]. Sanguinarine is a natural benzoanthidine alkaloid, isolated from the roots of *Draconis* and other medicinal *opium poppy*. Sanguinarine can adjust apoptosis, anti-proliferation, anti-angiogenesis and anti-invasion of skin, prostate, cervix, breast, blood system, gastrointestinal tract, pancreas and lung malignant tumors [8, 135]. In hepatocellular carcinoma, HIF-1α and TGF-β form a feedforward loop to induce EMT. Sanguinarine inhibits HIF-1α signaling and the expression of EMT markers, translocation of Snail and activation of both Smad and PI3K-AKT pathways to impair the proliferation of nine kinds of HCC cell lines and the colony formation of HCC cells [136]. SH is the main bioactive alkaloid isolated from *Sinomenium acutum*, and it inhibits the metastasis of human glioblastoma cells by inhibiting the activation of NF-κB and the expression of MMP-2/9 in vivo and in vitro, reversing endogenous and exogenous EMT and reversing the inflammatory microenvironment [137]. Berberine induces EMT changes in colonic epithelial cells with decreased E-cadherin and increased vimentin and α-SMA expression [2]. Triple negative breast cancer (TNBC) is a kind of breast cancer with the highest mutation, limited treatment options and poor prognosis [138]. BBR significantly decreases the level of TGF-β1 expression, but not TGF-β2 expression in TNBC cells to inhibit its growth and metastasis. The reduction of TGF-β1 expression by BBR treatment triggers suppression of cell migration through downregulation of MMP-2. Moreover, BBR also suppresses the phosphorylation level of Smad3 in TNBC cells [139]. TGF-β ligands signal via Smads and cooperating kinase pathways and control the expression or activities of key transcription factors that promote either epithelial differentiation or mesenchymal transitions. These Smad complexes mediate the transcription of multiple target genes related to EMT [140]. Halofuginone is a plant alkaloid derivative that inhibits phosphorylation of Smad proteins in response to TGF-β. And TGF-β induces Smad driven transcription to treat human melanoma cells, inhibiting cell proliferation, and it possesses antiangiogenic and anti-proliferative properties [141]. Neferine, a natural ingredient obtained from *lotus* seed, has anti-tumor effect, and it inhibits EMT-induced migration and invasion of cancer cells by up-regulating the expression of epithelial markers such as E-cadherin, and down-regulating the expression of stromal cell markers such as Vimentin, Snail and N-cadherin. Therefore, Neferine may play a certain anti-tumor effects by regulating the invasiveness and chemosensitivity of cancer cells through EMT [134]. Piperine reverses the biomarker expression of EMT, and inhibits colorectal cancer migratory and invasive capacities through STAT3/Snail mediated EMT [142]. An interesting study by Karolina Wojtowicz et analyzed the cytotoxic effect of piperine and cytostatic drugs throug MTT assay. Piperine increases the cytotoxic effect of paclitaxel and topotecan in drug-resistant cells [143]. (Fig. 6).

### Targeting extracellular matrix (ECM)

The recombination of extracellular matrix (ECM) is critical to the invasion of EMT and malignant cells. MMPs is the main component of the enzyme cascade of extracellular matrix degradation, cell–cell remodeling and cell–matrix interaction [134]. Tumor cells exist in the evolving interstitial microenvironment, and the vascular system, immune cells and tumor-related fibroblasts are all located in the dynamic extracellular matrix [144]. ECM is an acellular component of tumor microenvironment. The cellular components of tumor microenvironment can regulate the production and remodeling of extracellular matrix, provide physical and biochemical support for tumor cells, and promote tumor growth [145]. Morphine regulates the recruitment of tumor by immune cells and endothelial cells, and regulates the invasion and metastasis of tumor tissue by regulating extracellular matrix degrading enzymes, such as MMP-9 and urokinase-plasminogen activator(u-PA) through modulation of paracrine communication between cancer cells and nonmalignant cells in the tumor microenvironment [3]. Emetine is the main alkaloid of ipecac. Ipecac alkaloids are secondary metabolites produced in the medicinal plant *Psychotria ipecacuanha*. Modulation of three major MAPKs, ERK, p38 and JNK is well known to be involved in the regulation of MMPs, which are essential in tissue remodeling and ECM degradation, for ECM contributes to cancer cells spreading out from the origin of tumorigenesis. Emetine regulates two major MAPKs, p38 and ERK. This leads to the selective down-regulation of MMP-2 and MMP-9, two major gelatinases which can degrade ECM components [146, 147].

### Targeting reactive oxygen species (ROS)

Cancer cells and various tumor-associated stromal cells produce and secrete a copious amount of reactive oxygen species (ROS) into the TME and through many mechanisms that may be important for the treatment and prevention of cancer [148–152]. Soluble factors relevant for TME include oxygen, ROS, ATP, Ca2^+^, H^+^, growth factors or cytokines [153]. Although high levels of reactive oxygen species can lead to tissue damage and cell death, low levels of reactive oxygen species can produce proliferation [154, 155]. In different stages of tumor development, the effects of intracellular ROS on tumor cells are different. In the precancerous and early stages of tumor, intracellular ROS promote the occurrence of cancer by inducing oxidation and base pair substitution mutations in proto-oncogenes (such as Ras) and tumor suppressor genes (such as p53). With further development of the tumor, excessive accumulation of intracellular ROS will trigger apoptosis [11, 156, 157]. Hypoxia and the expression of HIF-1a and HIF-2a are characteristic of all solid tumors. HIFs suppress antitumor immune responses and promote tumor growth through direct growth-promoting cytokine production, angiogenesis, and ROS production. HIF signaling serves as a major adaptive mechanism in tumor growth in a hypoxic microenvironment [158]. Koumine, an alkaloid monomer found abundantly in Gelsemium plants, can be used as an effectively antioxidant. Koumine possesses the cytoprotective effects by suppressing production of ROS, inhibiting the caspase-3 activity and influencing the expression of Bax and Bcl-2 [159]. Capsaicin has multiple anticancer effects on many tumor cell lines. Capsaicin can regulate intracellular Ca^2+^ concentration and ROS- JNK-CCAAT/enhancer binding protein homologous protein pathway to inhibit cell proliferation, metastasis and induce apoptosis. We also found that it can abrogate LPS-induced NF-κB activation and it was noted that these inhibitory effects were mediated via its antioxidant activity [160]. In pancreatic cancer, Sophoridine can stimulate the production of ROS in pancreatic cancer cells, then activate ERK and JNK kinases, which trigger cell cycle arrest and mitochondrial apoptosis pathway in pancreatic cancer cells [161]. PANC-1 proliferates rapidly in tumor microenvironment under low blood volume and hypoxia conditions, showing a significant tolerance to nutritional starvation. A new 5-pyrrolidine-coupled naphthyl dihydroisoquinoline alkaloid isolated from Congovine *Ancistrocladus likoko* has significant priority cytotoxicity to PANC-1 cells under the condition of nutrient deficiency, thus inhibiting the growth of tumor cells [162].

### Alkaloids and extracellular vesicles (EVs)

Extracellular vesicles are the type of membrane debris produced by all cells, including lipid vesicles that transport proteins, lipids, DNA, mitochondrial DNA, messenger RNA, and microRNA. In recent years, extracellular vesicles have arisen as an important mechanism of cellular interchange of bioactive molecules. EVs isolated from tumor have been implicated in various steps of tumor progression, such as proliferation, angiogenesis, metastasis, and drug resistance [163, 164]. Songgang Gu etc. indicated that berberine inhibits cancer cell proliferation through decreasing secretion of extracellular vesicles. berberine is a promising candidate for the development of new therapies for cancer [165]. Xiao Du etc. showed Steroidal glycoalkaloids from Solanum lyratum exert anti-tumor angiogenesis by inhibiting the pro-angiogenic activity of A549-derived exosomes [166]. Qing Lin etc. Demonstrated a highly biocompatible tumor cell-targeting delivery systems utilizing exosome-like vesicles (ELVs) that delivers a low-toxicity anti-cancer agent imperialine against NSCLC [167]. EVs dynamically contribute to the heterogeneity of the tumor through their diverse cargo content. However, due to the lack of standardized isolation techniques that go beyond subcellular origin, size, and floatation density that the field still struggles to assess EVs heterogeneity. Further dissection of EVs heterogeneity will be requisite to enhancing our understanding of the critical roles of EVs in cancer [163].

## Conclusion

In conclusion, a large number of studies have demonstrated that the tumor microenvironment is closely associated with tumorigenesis and its progression. In recent years, people have become more and more aware of the important role of tumor microenvironment, which is like soil, providing a variety of conditions for the occurrence and development of tumor cells [168–170]. The majority of existing therapies have focused on the effect on the incipient cancer cell. However, the inhibition of biological programs that are associated with the tumor microenvironment may be critical to the prevention and treatment of cancer. Therefore, the consideration of the tumor microenvironment as a target for cancer prevention and treatment provides a unique perspective on both tumorigenesis and the therapy of cancer [171].

Alkaloids are a kind of natural monomer compounds extracted from traditional Chinese medicine, which have been reported to have significant anti-tumor effects. It can play its role by inhibiting telomerase activity, reducing tumor spread, inducing tumor cell differentiation and apoptosis, and inhibiting tumor cell cycle and proliferation [172–174]. However, there are still few studies on targeting alkaloids for tumor microenvironment, and most of the studies focused on in vitro and in vivo in laboratory. To strongly confirm the important role of traditional Chinese medicine and its monomer compounds in the anti-tumor process, it is necessary to conduct further experiments and clinical studies in vivo to describe its anti-tumor mechanism more completely. Combination therapy is an increasingly common method in clinical treatment. Alkaloids could serve as promising drugs in company with radiotherapy or chemotherapy agents and other types of immunotherapies, which may also present prospective opportunities to improve the quality of lifetime and survival of patients.

Recently, numerous studies have attempted to circumvent the limited in vivo activity and systemic toxicity of traditional Chinese medicines by incorporation into nanoparticle-based delivery systems, there are several challenges to achieving the full clinical potential of many alkoids, such as the high hydrophobicity and low bioavailability [175]. 10-Hydroxycamptothecin (10-HCPT), which is a promising anticancer drug with a wide spectrum of antitumor activities.10-HCPT nanocrystals prepared using modified acid-based microprecipitation and high pressure homogenization technology have been shown to enhance tumor drug accumulation and have better anticancer efficacy in mice [176]. With many different types of nanoparticles to choose the best type for a specific indication and optimize that particles properties such as size, shape, charge, material, surface functionalization, and choice of antigen and adjuvant, available as deliveryand targeting vehicles. Through these modifications like precise delivery with improved stability, biodistribution, pharmacokinetics, and toxicity profiles,it can be achieved as needed for clinical development [177].

Personalized medicine is patients can be selected for specific targeted therapies based on the molecular characteristics of the tumor and its microenvironment, allowing the right treatment to be delivered to the right person at the right time. Molecularly targeted agents have the potential to maximize antitumor efficacy while minimizing toxicity in cancer therapy. This emphasizes the significance of progressing personalized cancer therapeutic strategies [178]. Vintafolide is a water-soluble FR-targeting drug conjugate consisting of a folate moiety and desacetylvinblastine monohydrazide. Phase I studies showed a 2.5 mg bolus dose to be nontoxic, with moderately adverse events. Phase II clinical trials first showed a statistically significant improvement in disease-free survival in patients with platinum-resistant ovarian cancer. Phase III clinical trials are currently ongoing [179, 180]. Appropriately select and treat patients who are likely to benefit from specific targeted therapies, leading to amelioration in clinical and safety outcomes.

In summary, targeted therapy to tumor microenvironment will become the mainstream of cancer therapy with its unique advantages. Small molecule reagent derived from natural alkaloids by virtue of their powerful biological activity and exact therapeutic effect will also become a research hotspot in targeted therapy to tumor microenvironment. And with understanding of tumor pathology and the development of new drug research and development technology in depth, through structural modification of existing alkaloids, and utilized the nanotechnology and various modern technologies, the targeting of small molecule compounds will be improved, a promising candidates for the treatment of targeted tumor microenvironment is appeared, providing an alternative to cancer therapy. To lay the foundation for the further development of precision medicine, we firmly believe that the increasing of new anticancer drugs based on alkaloids targeting tumor microenvironment will be highly valued in the forthcoming future. Moreover, the combined application of alkaloid targeted reagents with tumor immunotherapy or clinical chemotherapeutic drugs is also expected to become a prospective research field.

## Data Availability

Not applicable.

## Abbreviations

TME: Tumor microenvironment
HGF: Hepatocyte growth factor
NLRP3: NLR family pyrin domain containing 3
CAFs: Cancer-associated fibroblasts
TGF-β: Transforming growth factor-β
CXCL12: C–X–C motif chemokine ligand 12
TIL: Tumor infiltrating lymphocyte
IL-6: Interleukin-6
CNP: Conophylline
CCL2: C–C motif chemokine ligand 2
OPN: Osteopontin
PD-L1: Programmed death ligand 1
TAMs: Tumor associated macrophages
Treg: Tumor-associated regulatory T cell
CTLA-4: Cytotoxic T lymphocyte-associated antigen-4
PD-1: Programmed cell death protein 1
DCs: Dendritic cells
SH: Stachydrine hydrochloride
NF-κB: Nuclear factor-κB
STAT3: Signal transducer and activator of transcription 3
TEBs: Tumor-educated B cells
RCC: Renal cell carcinoma
pDC: Plasmacytoid dendritic cells
IFN-α: Interferon-α
cDC: Conventional dendritic cells
OX40+pDC: Occurring pDC subgroup
MT: Matrine
OMT: Oxymatrine
HHT: Homoharringtonine
CLL: Chronic lymphocytic leukemia
COX-2: Cyclooxygenase-2
iNOs: Inducible nitric oxide synthase
TNF-α: Tumor necrosis factor-α
BBR: Berberine
EH: Ephedrine hydrochloride
MAPKs: Mitogen-activated protein kinases
ERKs: Extracellular signal-regulated kinases
JNKs: c-Jun N-terminal kinases
PGE2: Prostaglandin E2
PCX: Paclitaxel
MSCs: Mesenchymal stem cells
BMSCs: Bone marrow mesenchymal stem cell
PLGA: Paclitaxel (PTX)-poly (dpene-lactide-glycolide) nanoparticles
AML: Acute myeloid leukemia
CSCs: Cancer stem cells
MM: Multiple myeloma
LCL-HHT-H-PEG: Long-circulating liposomes containing HHT
MASM: (6aS,10S,11aR,11bR,11cS)-10-methylamino-dodecahydro-3a, 7a-diazabenzo(de)anthracene-hione
BCSCs: Breast cancer stem cells
BLE: Berberis libanotica Ehrenb
GBM: Glioblastom
GNPs: Granule neuron precursor
TAAs: Tumor-associated astrocytes
STA: Stachydrine
PSCs: Pancreatic stellate cells
PCCs: PANCREATIC cancer cells
VEGF: Vascular endothelial growth factor
HIF-1α: Hypoxia-inducible factor-1α
HIF-1: Hypoxia inducible factor
SH: Sinomenine hydrochloride
Evo: Evodiamine
CRC: Colorectal cancer
TNBC: Triple negative breast cancer
ECM: Extracellular matrix
u-PA: Urokinase-plasminogen activator
ROS: Reactive oxygen species
